# (1*R*,3*S*,5*R*,6*S*)-6-Acet­oxy-8-methyl-3-(*p*-tolyl­sulfon­yloxy)-8-azoniabicyclo­[3.2.1]octane (2*R*,3*R*)-2,3-bis­(benzo­yloxy)-3-carboxy­propanoate

**DOI:** 10.1107/S1600536809012732

**Published:** 2009-04-18

**Authors:** Li-Min Yang, Yi-Fan Xie, Ya-Fang Gu, Hong-Zhuan Chen, Yang Lu

**Affiliations:** a Department of Pharmacy, Shanghai Jiao Tong University School of Medicine, South Chongqing Road 280, Shanghai 200025, People’s Republic of China

## Abstract

The title compound, C_17_H_24_NO_5_S^+^·C_18_H_13_O_8_^−^, is the key inter­mediate during the preparation of lesatropane [systematic name (1*R*,3*S*,5*R*,6*S*)-6-acetoxy-3-(4-methylphenylsulfonyloxy)tropane], a potential anti­glaucoma agent. The tertiary N atom of the tropane ring is involved in inter­molecular N—H⋯O hydrogen bonding, and the carboxylate groups are involved in inter­molecular O—H⋯O hydrogen bonding.

## Related literature

For the crystal structure of lesatropane, see: Yang *et al.* (2008 ▶). For its improved agonistic activity compared to its racemic counterpart, see: Zhu *et al.* (2008 ▶). For synthetic details, see: Yang & Wang (1998 ▶).
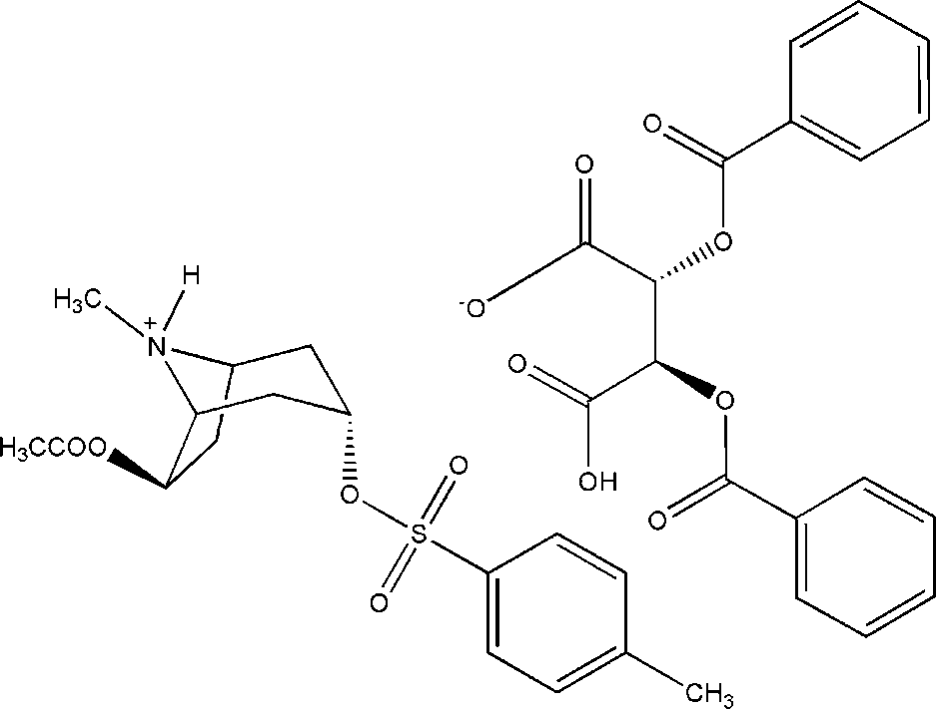


## Experimental

### 

#### Crystal data

C_17_H_24_NO_5_S^+^·C_18_H_13_O_8_^−^
*M* *_r_* = 711.72
Orthorhombic, 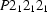

*a* = 7.4153 (5) Å
*b* = 19.2664 (12) Å
*c* = 24.7388 (16) Å
*V* = 3534.3 (4) Å^3^
*Z* = 4
Mo *K*α radiation
μ = 0.16 mm^−1^
*T* = 293 K
0.31 × 0.16 × 0.08 mm

#### Data collection

Bruker SMART CCD area-detector diffractometer
Absorption correction: multi-scan (**SADABS**; Sheldrick, 2002 ▶) *T*_min_ = 0.863, *T*_max_ = 1.000 (expected range = 0.852–0.987)
18779 measured reflections
6550 independent reflections
5135 reflections with *I* > 2σ(*I*)
*R*_int_ = 0.081

#### Refinement

*R*[*F* ^2^ > 2σ(*F* ^2^)] = 0.063
*wR*(*F* ^2^) = 0.125
*S* = 1.06
6550 reflections
459 parameters
1 restraint
H atoms treated by a mixture of independent and constrained refinement
Δρ_max_ = 0.19 e Å^−3^
Δρ_min_ = −0.17 e Å^−3^
Absolute structure: Flack (1983 ▶), 3094 Friedel pairs
Flack parameter: 0.03 (12)

### 

Data collection: *SMART* (Bruker, 2001 ▶); cell refinement: *SAINT* (Bruker, 2001 ▶); data reduction: *SAINT*; program(s) used to solve structure: *SHELXS97* (Sheldrick, 2008 ▶); program(s) used to refine structure: *SHELXL97* (Sheldrick, 2008 ▶); molecular graphics: *SHELXTL* (Sheldrick, 2008 ▶); software used to prepare material for publication: *SHELXTL*.

## Supplementary Material

Crystal structure: contains datablocks I, global. DOI: 10.1107/S1600536809012732/ww2139sup1.cif

Structure factors: contains datablocks I. DOI: 10.1107/S1600536809012732/ww2139Isup2.hkl

Additional supplementary materials:  crystallographic information; 3D view; checkCIF report

## Figures and Tables

**Table 1:** Hydrogen-bond geometry (Å, °)

*D*—H⋯*A*	*D*—H	H⋯*A*	*D*⋯*A*	*D*—H⋯*A*
N1—H1*A*⋯O1^i^	0.860 (18)	1.89 (2)	2.699 (4)	156 (3)
O4—H4⋯O2^i^	0.82	1.66	2.460 (3)	164

Symmetry code: (i) 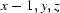.

**Fig. 1.**
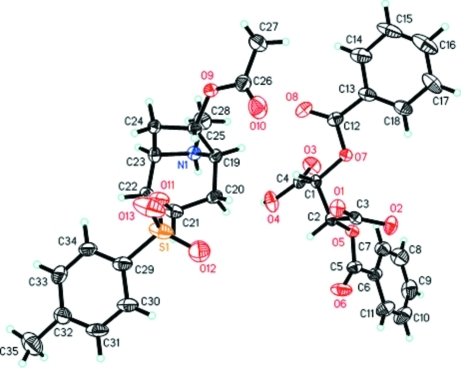
The molecular structure of the title compound with displacement ellipsoids drawn at the 50% probability level.

**Fig. 2.**
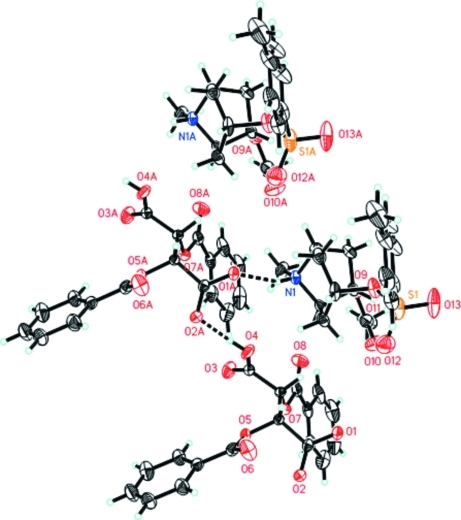
The intermolecular N–H···O and O–H···O hydrogen bonds (dashed lines).

## supplementary crystallographic information

### Comment

6β-Acetoxy-3α-paramethylbenzene sulfonyloxy tropane is a potent muscarinic receptor agonist and has been shown to be a promising candidate as a new antiglaucoma agent. The pharmacology results suggest that the (1*R*,3*S*,5*R*,6*S*) isomer (lesatropane), the crystal structure has been reported (Yang *et al.*, 2008), displays an improved agonistic activity compared to its racemic counterpart (Zhu *et al.*, 2008). The enantiopure isomer was obtained by the optical resolution of the racemic tropane alkaloids with the chiral acid (Yang & Wang, 1998). We report here the crystal structure of the diastereoisomeric salt, (1*R*,3*S*,5*R*,6*S*)-6-acetoxy-3-paramethylbenzene sulfonyloxytropane and (-)-*O*',*O*'-dibenzyl-*L*-tartaric acid (1/1), formed during the resolution. The three-dimensional structure of the title compound is shown in Fig.1. X-ray structure analytical data showed that the diastereoisomeric salt is produced by the formation of hydrogen bonds. The nitrogen atom of the tropane alkaloid is protonated to form the cation and the chiral acid is deprotonated to form anion. Each anion interacts with a cation (*via* N atom) forming N–H···O hydrogen bond, and chiral acid anions are linked by O–H···O hydrogen bond with each other (Fig. 2).

### Experimental

Rac 6β-acetoxy-3α-paramethylbenzene sulfonyloxytropane (586.3 mg, 1.66 mmol) and (-)-2,3-dibenzoyl-*L*-tartaric acid (728.3 mg, 2.03 mmol) were dissolved in methanol. After disposing at room temperature for 12 h, the title compound as precipitate was collected by filtration. Three recrystallizations of the crude product from anhydrous ethanol gave pure colorless crystals, 30% yield, m.p. 443–445 K, [α]_D_^20^ -14.23 (c = 0.084, EtOH).

### Refinement

H atoms were located in a difference Fourier map and refined isotropically with bond restraint: N1–H1A=0.860 (18)Å, other H atoms were positioned geometrically and treated as riding, with C–H and O–H bond lengths constrained to 0.96Å for methyl, 0.97Å for methylene, 0.98Å for methine, 0.93Å for C*sp*^2^—H and 0.82Å for hydroxyl, with *U*_iso_(H) = 1.5*U*_eq_(methyl C and hydroxyl O) and *U*_iso_(H) = 1.2*U*_eq_(methylene and methine C). The 3094 Friedel pairs were used in the measurement of the Flack parameter (Flack, 1983).

### Crystal data

**Table:**

C_17_H_24_NO_5_S^+^·C_18_H_13_O_8_^−^	*D*_x_ = 1.338 Mg m^−^^3^
*M**_r_* = 711.72	Melting point: 445 K
Orthorhombic, *P*2_1_2_1_2_1_	Mo *K*α radiation, λ = 0.71073 Å
Hall symbol: P 2ac 2ab	Cell parameters from 3212 reflections
*a* = 7.4153 (5) Å	θ = 4.5–39.6°
*b* = 19.2664 (12) Å	µ = 0.16 mm^−^^1^
*c* = 24.7388 (16) Å	*T* = 293 K
*V* = 3534.3 (4) Å^3^	Prismatic, colorless
*Z* = 4	0.31 × 0.16 × 0.08 mm
*F*(000) = 1496	

### Data collection

**Table:**

Bruker SMART CCD area-detector diffractometer	6550 independent reflections
Radiation source: fine-focus sealed tube	5135 reflections with *I* > 2σ(*I*)
graphite	*R*_int_ = 0.081
φ and ω scans	θ_max_ = 25.5°, θ_min_ = 1.7°
Absorption correction: multi-scan (*SADABS*; Sheldrick, 2002)	*h* = −8→8
*T*_min_ = 0.863, *T*_max_ = 1.000	*k* = −23→22
18779 measured reflections	*l* = −29→25

### Refinement

**Table:**

Refinement on *F*^2^	Secondary atom site location: difference Fourier map
Least-squares matrix: full	Hydrogen site location: inferred from neighbouring sites
*R*[*F*^2^ > 2σ(*F*^2^)] = 0.063	H atoms treated by a mixture of independent and constrained refinement
*wR*(*F*^2^) = 0.125	*w* = 1/[σ^2^(*F*_o_^2^) + (0.0406*P*)^2^] where *P* = (*F*_o_^2^ + 2*F*_c_^2^)/3
*S* = 1.06	(Δ/σ)_max_ = 0.002
6550 reflections	Δρ_max_ = 0.19 e Å^−^^3^
459 parameters	Δρ_min_ = −0.17 e Å^−^^3^
1 restraint	Absolute structure: Flack (1983), 3094 Friedel pairs
Primary atom site location: structure-invariant direct methods	Flack parameter: 0.03 (12)

### Special details

**Table:**

Geometry. All e.s.d.'s (except the e.s.d. in the dihedral angle between two l.s. planes) are estimated using the full covariance matrix. The cell e.s.d.'s are taken into account individually in the estimation of e.s.d.'s in distances, angles and torsion angles; correlations between e.s.d.'s in cell parameters are only used when they are defined by crystal symmetry. An approximate (isotropic) treatment of cell e.s.d.'s is used for estimating e.s.d.'s involving l.s. planes.
Refinement. Refinement of *F*^2^ against ALL reflections. The weighted *R*-factor *wR* and goodness of fit *S* are based on *F*^2^, conventional *R*-factors *R* are based on *F*, with *F* set to zero for negative *F*^2^. The threshold expression of *F*^2^ > σ(*F*^2^) is used only for calculating *R*-factors(gt) *etc*. and is not relevant to the choice of reflections for refinement. *R*-factors based on *F*^2^ are statistically about twice as large as those based on *F*, and *R*- factors based on ALL data will be even larger.

### Fractional atomic coordinates and isotropic or equivalent isotropic displacement parameters (Å^2^)

**Table:**

	*x*	*y*	*z*	*U*_iso_*/*U*_eq_	
S1	0.43913 (19)	0.80222 (5)	0.07318 (5)	0.0665 (4)	
O1	0.8630 (3)	0.65740 (12)	0.25014 (9)	0.0447 (6)	
O2	0.9488 (3)	0.64372 (13)	0.33606 (9)	0.0503 (7)	
O3	0.3089 (4)	0.53125 (14)	0.33986 (11)	0.0635 (8)	
O4	0.2542 (3)	0.63195 (15)	0.29838 (11)	0.0607 (8)	
H4	0.1541	0.6282	0.3121	0.091*	
O5	0.6110 (3)	0.64586 (10)	0.37312 (8)	0.0344 (5)	
O6	0.6373 (5)	0.76048 (13)	0.37990 (11)	0.0713 (9)	
O7	0.6535 (3)	0.52908 (11)	0.29852 (8)	0.0381 (6)	
O8	0.4990 (4)	0.47399 (14)	0.23402 (11)	0.0665 (8)	
O9	0.3342 (3)	0.51778 (11)	0.09819 (9)	0.0437 (6)	
O10	0.6199 (4)	0.54125 (18)	0.11767 (14)	0.0864 (10)	
O11	0.3041 (4)	0.74042 (11)	0.08365 (9)	0.0567 (8)	
O12	0.5495 (5)	0.81346 (16)	0.12012 (13)	0.0827 (10)	
O13	0.5200 (6)	0.78393 (14)	0.02309 (13)	0.1013 (14)	
N1	0.0691 (4)	0.60385 (15)	0.17050 (12)	0.0403 (7)	
C1	0.5418 (4)	0.58904 (15)	0.28989 (13)	0.0312 (7)	
H1	0.5325	0.5974	0.2509	0.037*	
C2	0.6353 (4)	0.65030 (16)	0.31564 (11)	0.0308 (7)	
H2	0.5784	0.6931	0.3026	0.037*	
C3	0.8340 (4)	0.65123 (16)	0.29961 (14)	0.0337 (8)	
C4	0.3536 (5)	0.57930 (19)	0.31248 (13)	0.0382 (8)	
C5	0.6121 (5)	0.70611 (18)	0.40081 (14)	0.0425 (9)	
C6	0.5822 (5)	0.69397 (18)	0.45963 (14)	0.0431 (9)	
C7	0.5218 (6)	0.6320 (2)	0.48022 (15)	0.0561 (11)	
H7	0.5015	0.5949	0.4570	0.067*	
C8	0.4907 (7)	0.6240 (2)	0.53502 (18)	0.0750 (14)	
H8	0.4476	0.5821	0.5485	0.090*	
C9	0.5241 (7)	0.6785 (3)	0.56931 (18)	0.0791 (15)	
H9	0.5055	0.6732	0.6063	0.095*	
C10	0.5841 (7)	0.7403 (3)	0.54952 (18)	0.0780 (15)	
H10	0.6052	0.7771	0.5730	0.094*	
C11	0.6139 (6)	0.7486 (2)	0.49489 (17)	0.0663 (12)	
H11	0.6553	0.7909	0.4817	0.080*	
C12	0.6235 (5)	0.47548 (18)	0.26441 (14)	0.0431 (9)	
C13	0.7706 (6)	0.42341 (17)	0.26702 (14)	0.0464 (10)	
C14	0.7538 (7)	0.36354 (19)	0.23575 (15)	0.0697 (14)	
H14	0.6463	0.3543	0.2178	0.084*	
C15	0.8981 (10)	0.3175 (2)	0.2315 (2)	0.0876 (19)	
H15	0.8885	0.2781	0.2100	0.105*	
C16	1.0522 (10)	0.3306 (3)	0.2589 (2)	0.093 (2)	
H16	1.1479	0.2996	0.2560	0.111*	
C17	1.0715 (7)	0.3883 (2)	0.2910 (2)	0.0732 (14)	
H17	1.1785	0.3962	0.3096	0.088*	
C18	0.9293 (6)	0.43443 (18)	0.29510 (16)	0.0529 (11)	
H18	0.9405	0.4734	0.3170	0.064*	
C19	0.2672 (5)	0.61242 (17)	0.16238 (12)	0.0380 (8)	
H19	0.3349	0.5816	0.1865	0.046*	
C20	0.3139 (5)	0.68767 (17)	0.17330 (13)	0.0448 (9)	
H20A	0.4423	0.6944	0.1680	0.054*	
H20B	0.2865	0.6984	0.2107	0.054*	
C21	0.2105 (6)	0.73772 (18)	0.13658 (14)	0.0509 (11)	
H21	0.2102	0.7841	0.1529	0.061*	
C22	0.0196 (6)	0.71503 (18)	0.12643 (15)	0.0533 (10)	
H22A	−0.0239	0.7379	0.0940	0.064*	
H22B	−0.0548	0.7305	0.1563	0.064*	
C23	−0.0048 (5)	0.63679 (18)	0.11971 (14)	0.0450 (9)	
H23	−0.1322	0.6249	0.1146	0.054*	
C24	0.1113 (5)	0.60421 (18)	0.07602 (13)	0.0459 (10)	
H24A	0.1233	0.6353	0.0454	0.055*	
H24B	0.0591	0.5608	0.0637	0.055*	
C25	0.2953 (5)	0.59160 (16)	0.10286 (13)	0.0365 (8)	
H25	0.3898	0.6197	0.0859	0.044*	
C26	0.5068 (6)	0.4997 (2)	0.10702 (16)	0.0547 (11)	
C27	0.5346 (8)	0.4234 (2)	0.10171 (19)	0.0869 (16)	
H27A	0.6415	0.4101	0.1210	0.130*	
H27B	0.5478	0.4116	0.0642	0.130*	
H27C	0.4325	0.3993	0.1165	0.130*	
C28	0.0126 (6)	0.53036 (19)	0.17885 (16)	0.0608 (11)	
H28A	0.0494	0.5030	0.1484	0.091*	
H28B	−0.1162	0.5282	0.1825	0.091*	
H28C	0.0682	0.5126	0.2110	0.091*	
C29	0.2935 (7)	0.87247 (18)	0.06412 (14)	0.0555 (11)	
C30	0.3384 (7)	0.93614 (18)	0.08560 (15)	0.0591 (12)	
H30	0.4453	0.9419	0.1047	0.071*	
C31	0.2217 (8)	0.9912 (2)	0.07822 (17)	0.0699 (14)	
H31	0.2523	1.0342	0.0925	0.084*	
C32	0.0617 (8)	0.9846 (2)	0.05054 (16)	0.0696 (14)	
C33	0.0230 (9)	0.9193 (2)	0.03008 (17)	0.0857 (17)	
H33	−0.0847	0.9128	0.0115	0.103*	
C34	0.1363 (8)	0.8643 (2)	0.03609 (16)	0.0794 (17)	
H34	0.1069	0.8214	0.0212	0.095*	
C35	−0.0664 (10)	1.0455 (3)	0.04588 (19)	0.111 (2)	
H35A	−0.1094	1.0580	0.0812	0.167*	
H35B	−0.1667	1.0329	0.0234	0.167*	
H35C	−0.0043	1.0843	0.0301	0.167*	
H1A	0.032 (5)	0.6269 (16)	0.1981 (11)	0.054 (12)*	

### Atomic displacement parameters (Å^2^)

**Table:**

	*U*^11^	*U*^22^	*U*^33^	*U*^12^	*U*^13^	*U*^23^
S1	0.0980 (10)	0.0401 (5)	0.0614 (7)	−0.0053 (6)	0.0372 (7)	0.0059 (5)
O1	0.0397 (14)	0.0591 (15)	0.0352 (13)	−0.0003 (13)	0.0084 (11)	0.0051 (11)
O2	0.0320 (14)	0.0785 (18)	0.0404 (14)	0.0036 (14)	0.0004 (12)	−0.0020 (13)
O3	0.0570 (19)	0.0574 (17)	0.076 (2)	−0.0078 (15)	0.0169 (15)	0.0155 (15)
O4	0.0278 (14)	0.087 (2)	0.0668 (18)	0.0102 (15)	0.0113 (13)	0.0265 (16)
O5	0.0368 (13)	0.0345 (12)	0.0318 (12)	0.0018 (11)	0.0078 (10)	−0.0011 (10)
O6	0.121 (3)	0.0339 (14)	0.0589 (18)	−0.0120 (16)	0.0077 (18)	−0.0011 (13)
O7	0.0399 (14)	0.0359 (12)	0.0385 (13)	0.0052 (11)	−0.0030 (11)	−0.0047 (10)
O8	0.075 (2)	0.0667 (18)	0.0578 (18)	−0.0039 (17)	−0.0186 (16)	−0.0172 (15)
O9	0.0515 (17)	0.0386 (13)	0.0409 (14)	0.0016 (12)	−0.0038 (12)	−0.0042 (11)
O10	0.053 (2)	0.086 (2)	0.120 (3)	−0.0026 (19)	−0.015 (2)	0.012 (2)
O11	0.095 (2)	0.0355 (13)	0.0397 (15)	−0.0029 (14)	0.0216 (14)	−0.0002 (11)
O12	0.077 (2)	0.077 (2)	0.093 (2)	−0.0141 (19)	−0.003 (2)	0.0102 (18)
O13	0.159 (4)	0.0518 (18)	0.093 (2)	0.002 (2)	0.081 (2)	0.0111 (16)
N1	0.0452 (19)	0.0415 (17)	0.0341 (17)	−0.0024 (14)	0.0082 (14)	0.0053 (14)
C1	0.0264 (18)	0.0359 (16)	0.0314 (17)	0.0040 (15)	−0.0011 (14)	0.0034 (14)
C2	0.0279 (18)	0.0348 (17)	0.0299 (17)	0.0031 (15)	0.0025 (14)	0.0021 (14)
C3	0.0327 (19)	0.0298 (16)	0.038 (2)	0.0031 (15)	0.0062 (16)	0.0033 (15)
C4	0.034 (2)	0.048 (2)	0.0325 (19)	−0.0028 (18)	0.0006 (16)	0.0029 (16)
C5	0.039 (2)	0.041 (2)	0.047 (2)	−0.0050 (18)	0.0061 (17)	−0.0086 (18)
C6	0.037 (2)	0.047 (2)	0.045 (2)	−0.0001 (18)	0.0039 (17)	−0.0130 (18)
C7	0.067 (3)	0.058 (2)	0.044 (2)	0.007 (2)	0.007 (2)	−0.0068 (19)
C8	0.093 (4)	0.078 (3)	0.054 (3)	0.010 (3)	0.015 (3)	0.007 (2)
C9	0.084 (4)	0.115 (4)	0.038 (3)	0.016 (3)	0.002 (2)	0.001 (3)
C10	0.074 (4)	0.106 (4)	0.054 (3)	0.001 (3)	0.002 (3)	−0.037 (3)
C11	0.073 (3)	0.075 (3)	0.050 (3)	−0.013 (3)	0.007 (2)	−0.020 (2)
C12	0.053 (2)	0.045 (2)	0.031 (2)	−0.0080 (19)	0.0071 (18)	−0.0013 (17)
C13	0.074 (3)	0.0310 (18)	0.035 (2)	0.0024 (19)	0.018 (2)	0.0075 (16)
C14	0.123 (4)	0.046 (2)	0.040 (2)	0.006 (3)	0.020 (2)	0.0053 (19)
C15	0.158 (6)	0.039 (2)	0.066 (3)	0.033 (3)	0.036 (4)	0.004 (2)
C16	0.132 (6)	0.057 (3)	0.090 (4)	0.038 (4)	0.054 (4)	0.026 (3)
C17	0.075 (3)	0.055 (3)	0.089 (3)	0.023 (2)	0.025 (3)	0.026 (2)
C18	0.063 (3)	0.039 (2)	0.057 (2)	0.004 (2)	0.020 (2)	0.0065 (18)
C19	0.048 (2)	0.0395 (18)	0.0269 (18)	−0.0054 (17)	−0.0006 (16)	0.0074 (15)
C20	0.061 (3)	0.043 (2)	0.0304 (19)	−0.0098 (19)	−0.0002 (17)	−0.0058 (16)
C21	0.075 (3)	0.0370 (19)	0.041 (2)	−0.002 (2)	0.026 (2)	0.0010 (16)
C22	0.066 (3)	0.050 (2)	0.043 (2)	0.013 (2)	0.006 (2)	0.0064 (18)
C23	0.037 (2)	0.053 (2)	0.045 (2)	0.0005 (18)	−0.0034 (17)	0.0060 (18)
C24	0.063 (3)	0.0428 (19)	0.0320 (19)	0.0073 (19)	−0.0083 (18)	0.0002 (16)
C25	0.048 (2)	0.0293 (17)	0.0320 (19)	−0.0012 (17)	0.0033 (16)	0.0030 (14)
C26	0.053 (3)	0.062 (3)	0.049 (2)	0.003 (2)	−0.002 (2)	−0.003 (2)
C27	0.114 (5)	0.066 (3)	0.081 (3)	0.034 (3)	−0.019 (3)	−0.010 (3)
C28	0.060 (3)	0.056 (2)	0.066 (3)	−0.013 (2)	0.013 (2)	0.020 (2)
C29	0.099 (4)	0.039 (2)	0.028 (2)	−0.012 (2)	0.013 (2)	−0.0027 (16)
C30	0.094 (4)	0.037 (2)	0.047 (2)	−0.015 (2)	0.013 (2)	−0.0071 (17)
C31	0.127 (5)	0.037 (2)	0.045 (3)	−0.012 (3)	0.017 (3)	−0.0121 (19)
C32	0.123 (5)	0.052 (3)	0.034 (2)	0.006 (3)	0.000 (3)	0.0035 (19)
C33	0.141 (5)	0.068 (3)	0.048 (3)	−0.005 (3)	−0.037 (3)	0.004 (2)
C34	0.157 (5)	0.035 (2)	0.047 (3)	−0.011 (3)	−0.026 (3)	0.0005 (19)
C35	0.183 (7)	0.086 (4)	0.064 (3)	0.055 (4)	0.009 (4)	0.004 (3)

### Geometric parameters (Å, °)

**Table:**

S1—O13	1.421 (3)	C15—C16	1.353 (7)
S1—O12	1.437 (3)	C15—H15	0.9300
S1—O11	1.577 (3)	C16—C17	1.372 (7)
S1—C29	1.746 (4)	C16—H16	0.9300
O1—C3	1.248 (4)	C17—C18	1.383 (5)
O2—C3	1.249 (4)	C17—H17	0.9300
O3—C4	1.194 (4)	C18—H18	0.9300
O4—C4	1.301 (4)	C19—C20	1.515 (5)
O4—H4	0.8200	C19—C25	1.540 (4)
O5—C5	1.348 (4)	C19—H19	0.9800
O5—C2	1.436 (3)	C20—C21	1.530 (5)
O6—C5	1.183 (4)	C20—H20A	0.9700
O7—C12	1.352 (4)	C20—H20B	0.9700
O7—C1	1.437 (3)	C21—C22	1.503 (6)
O8—C12	1.191 (4)	C21—H21	0.9800
O9—C26	1.344 (5)	C22—C23	1.527 (5)
O9—C25	1.456 (4)	C22—H22A	0.9700
O10—C26	1.189 (5)	C22—H22B	0.9700
O11—C21	1.483 (4)	C23—C24	1.518 (5)
N1—C28	1.491 (4)	C23—H23	0.9800
N1—C19	1.492 (5)	C24—C25	1.537 (5)
N1—C23	1.511 (4)	C24—H24A	0.9700
N1—H1A	0.860 (18)	C24—H24B	0.9700
C1—C2	1.510 (4)	C25—H25	0.9800
C1—C4	1.515 (5)	C26—C27	1.490 (5)
C1—H1	0.9800	C27—H27A	0.9600
C2—C3	1.525 (4)	C27—H27B	0.9600
C2—H2	0.9800	C27—H27C	0.9600
C5—C6	1.491 (5)	C28—H28A	0.9600
C6—C7	1.373 (5)	C28—H28B	0.9600
C6—C11	1.387 (5)	C28—H28C	0.9600
C7—C8	1.384 (5)	C29—C34	1.365 (6)
C7—H7	0.9300	C29—C30	1.378 (5)
C8—C9	1.373 (6)	C30—C31	1.381 (6)
C8—H8	0.9300	C30—H30	0.9300
C9—C10	1.362 (6)	C31—C32	1.375 (7)
C9—H9	0.9300	C31—H31	0.9300
C10—C11	1.379 (6)	C32—C33	1.386 (6)
C10—H10	0.9300	C32—C35	1.515 (7)
C11—H11	0.9300	C33—C34	1.360 (7)
C12—C13	1.484 (5)	C33—H33	0.9300
C13—C18	1.383 (5)	C34—H34	0.9300
C13—C14	1.394 (5)	C35—H35A	0.9600
C14—C15	1.393 (7)	C35—H35B	0.9600
C14—H14	0.9300	C35—H35C	0.9600
			
O13—S1—O12	120.1 (2)	C20—C19—C25	112.9 (3)
O13—S1—O11	102.92 (17)	N1—C19—H19	110.8
O12—S1—O11	110.05 (17)	C20—C19—H19	110.8
O13—S1—C29	109.95 (19)	C25—C19—H19	110.8
O12—S1—C29	109.84 (19)	C19—C20—C21	112.5 (3)
O11—S1—C29	102.33 (19)	C19—C20—H20A	109.1
C4—O4—H4	109.5	C21—C20—H20A	109.1
C5—O5—C2	116.8 (2)	C19—C20—H20B	109.1
C12—O7—C1	115.2 (3)	C21—C20—H20B	109.1
C26—O9—C25	115.4 (3)	H20A—C20—H20B	107.8
C21—O11—S1	117.9 (2)	O11—C21—C22	107.7 (3)
C28—N1—C19	113.6 (3)	O11—C21—C20	108.2 (3)
C28—N1—C23	114.4 (3)	C22—C21—C20	112.8 (3)
C19—N1—C23	101.5 (3)	O11—C21—H21	109.4
C28—N1—H1A	107 (2)	C22—C21—H21	109.4
C19—N1—H1A	111 (3)	C20—C21—H21	109.4
C23—N1—H1A	109 (2)	C21—C22—C23	114.6 (3)
O7—C1—C2	107.5 (2)	C21—C22—H22A	108.6
O7—C1—C4	112.1 (3)	C23—C22—H22A	108.6
C2—C1—C4	111.4 (3)	C21—C22—H22B	108.6
O7—C1—H1	108.6	C23—C22—H22B	108.6
C2—C1—H1	108.6	H22A—C22—H22B	107.6
C4—C1—H1	108.6	N1—C23—C24	102.3 (3)
O5—C2—C1	108.3 (2)	N1—C23—C22	106.3 (3)
O5—C2—C3	112.3 (3)	C24—C23—C22	114.7 (3)
C1—C2—C3	110.1 (3)	N1—C23—H23	111.0
O5—C2—H2	108.7	C24—C23—H23	111.0
C1—C2—H2	108.7	C22—C23—H23	111.0
C3—C2—H2	108.7	C23—C24—C25	105.2 (3)
O1—C3—O2	127.0 (3)	C23—C24—H24A	110.7
O1—C3—C2	115.0 (3)	C25—C24—H24A	110.7
O2—C3—C2	118.0 (3)	C23—C24—H24B	110.7
O3—C4—O4	126.8 (3)	C25—C24—H24B	110.7
O3—C4—C1	124.1 (3)	H24A—C24—H24B	108.8
O4—C4—C1	109.0 (3)	O9—C25—C24	107.2 (3)
O6—C5—O5	122.8 (3)	O9—C25—C19	110.9 (2)
O6—C5—C6	126.1 (3)	C24—C25—C19	104.6 (3)
O5—C5—C6	111.1 (3)	O9—C25—H25	111.3
C7—C6—C11	118.8 (4)	C24—C25—H25	111.3
C7—C6—C5	123.2 (3)	C19—C25—H25	111.3
C11—C6—C5	118.0 (4)	O10—C26—O9	122.2 (4)
C6—C7—C8	121.0 (4)	O10—C26—C27	125.9 (4)
C6—C7—H7	119.5	O9—C26—C27	111.9 (4)
C8—C7—H7	119.5	C26—C27—H27A	109.5
C9—C8—C7	119.3 (4)	C26—C27—H27B	109.5
C9—C8—H8	120.3	H27A—C27—H27B	109.5
C7—C8—H8	120.3	C26—C27—H27C	109.5
C10—C9—C8	120.4 (4)	H27A—C27—H27C	109.5
C10—C9—H9	119.8	H27B—C27—H27C	109.5
C8—C9—H9	119.8	N1—C28—H28A	109.5
C9—C10—C11	120.4 (4)	N1—C28—H28B	109.5
C9—C10—H10	119.8	H28A—C28—H28B	109.5
C11—C10—H10	119.8	N1—C28—H28C	109.5
C10—C11—C6	120.1 (4)	H28A—C28—H28C	109.5
C10—C11—H11	119.9	H28B—C28—H28C	109.5
C6—C11—H11	119.9	C34—C29—C30	120.3 (4)
O8—C12—O7	122.7 (4)	C34—C29—S1	120.3 (3)
O8—C12—C13	125.5 (3)	C30—C29—S1	119.4 (4)
O7—C12—C13	111.6 (3)	C29—C30—C31	118.8 (4)
C18—C13—C14	118.8 (4)	C29—C30—H30	120.6
C18—C13—C12	122.9 (3)	C31—C30—H30	120.6
C14—C13—C12	118.0 (4)	C32—C31—C30	122.4 (4)
C15—C14—C13	120.0 (5)	C32—C31—H31	118.8
C15—C14—H14	120.0	C30—C31—H31	118.8
C13—C14—H14	120.0	C31—C32—C33	116.4 (5)
C16—C15—C14	119.5 (5)	C31—C32—C35	120.5 (4)
C16—C15—H15	120.3	C33—C32—C35	123.1 (5)
C14—C15—H15	120.3	C34—C33—C32	122.6 (5)
C15—C16—C17	121.9 (5)	C34—C33—H33	118.7
C15—C16—H16	119.0	C32—C33—H33	118.7
C17—C16—H16	119.0	C33—C34—C29	119.5 (4)
C16—C17—C18	118.9 (5)	C33—C34—H34	120.2
C16—C17—H17	120.5	C29—C34—H34	120.2
C18—C17—H17	120.5	C32—C35—H35A	109.5
C13—C18—C17	120.9 (4)	C32—C35—H35B	109.5
C13—C18—H18	119.6	H35A—C35—H35B	109.5
C17—C18—H18	119.6	C32—C35—H35C	109.5
N1—C19—C20	107.9 (3)	H35A—C35—H35C	109.5
N1—C19—C25	103.5 (3)	H35B—C35—H35C	109.5
			
O13—S1—O11—C21	172.7 (3)	C28—N1—C19—C20	−161.2 (3)
O12—S1—O11—C21	43.5 (3)	C23—N1—C19—C20	75.6 (3)
C29—S1—O11—C21	−73.2 (3)	C28—N1—C19—C25	79.0 (3)
C12—O7—C1—C2	−159.4 (3)	C23—N1—C19—C25	−44.2 (3)
C12—O7—C1—C4	77.9 (3)	N1—C19—C20—C21	−58.4 (4)
C5—O5—C2—C1	−152.1 (3)	C25—C19—C20—C21	55.3 (4)
C5—O5—C2—C3	86.1 (3)	S1—O11—C21—C22	140.3 (3)
O7—C1—C2—O5	−76.2 (3)	S1—O11—C21—C20	−97.5 (3)
C4—C1—C2—O5	47.0 (3)	C19—C20—C21—O11	−81.1 (4)
O7—C1—C2—C3	47.0 (3)	C19—C20—C21—C22	37.9 (4)
C4—C1—C2—C3	170.1 (3)	O11—C21—C22—C23	81.2 (4)
O5—C2—C3—O1	−177.7 (3)	C20—C21—C22—C23	−38.1 (4)
C1—C2—C3—O1	61.6 (4)	C28—N1—C23—C24	−75.5 (4)
O5—C2—C3—O2	4.5 (4)	C19—N1—C23—C24	47.2 (3)
C1—C2—C3—O2	−116.2 (3)	C28—N1—C23—C22	163.8 (3)
O7—C1—C4—O3	8.4 (5)	C19—N1—C23—C22	−73.5 (3)
C2—C1—C4—O3	−112.1 (4)	C21—C22—C23—N1	56.9 (4)
O7—C1—C4—O4	−173.3 (3)	C21—C22—C23—C24	−55.3 (4)
C2—C1—C4—O4	66.2 (3)	N1—C23—C24—C25	−31.6 (3)
C2—O5—C5—O6	−2.3 (5)	C22—C23—C24—C25	83.1 (4)
C2—O5—C5—C6	179.0 (3)	C26—O9—C25—C24	164.0 (3)
O6—C5—C6—C7	167.8 (4)	C26—O9—C25—C19	−82.3 (4)
O5—C5—C6—C7	−13.5 (5)	C23—C24—C25—O9	122.5 (3)
O6—C5—C6—C11	−10.9 (6)	C23—C24—C25—C19	4.7 (3)
O5—C5—C6—C11	167.8 (3)	N1—C19—C25—O9	−90.9 (3)
C11—C6—C7—C8	0.8 (6)	C20—C19—C25—O9	152.8 (3)
C5—C6—C7—C8	−177.9 (4)	N1—C19—C25—C24	24.4 (3)
C6—C7—C8—C9	−1.2 (7)	C20—C19—C25—C24	−92.0 (3)
C7—C8—C9—C10	1.2 (8)	C25—O9—C26—O10	−0.3 (6)
C8—C9—C10—C11	−0.7 (8)	C25—O9—C26—C27	179.7 (3)
C9—C10—C11—C6	0.2 (8)	O13—S1—C29—C34	69.6 (4)
C7—C6—C11—C10	−0.3 (7)	O12—S1—C29—C34	−156.1 (3)
C5—C6—C11—C10	178.5 (4)	O11—S1—C29—C34	−39.3 (4)
C1—O7—C12—O8	−9.3 (5)	O13—S1—C29—C30	−110.6 (3)
C1—O7—C12—C13	166.2 (3)	O12—S1—C29—C30	23.7 (4)
O8—C12—C13—C18	166.6 (4)	O11—S1—C29—C30	140.6 (3)
O7—C12—C13—C18	−8.7 (5)	C34—C29—C30—C31	0.0 (6)
O8—C12—C13—C14	−7.5 (5)	S1—C29—C30—C31	−179.8 (3)
O7—C12—C13—C14	177.1 (3)	C29—C30—C31—C32	0.5 (6)
C18—C13—C14—C15	−2.6 (5)	C30—C31—C32—C33	−0.1 (7)
C12—C13—C14—C15	171.8 (4)	C30—C31—C32—C35	176.9 (4)
C13—C14—C15—C16	1.6 (7)	C31—C32—C33—C34	−0.8 (7)
C14—C15—C16—C17	−0.2 (8)	C35—C32—C33—C34	−177.7 (5)
C15—C16—C17—C18	−0.3 (7)	C32—C33—C34—C29	1.2 (8)
C14—C13—C18—C17	2.2 (5)	C30—C29—C34—C33	−0.9 (7)
C12—C13—C18—C17	−171.9 (3)	S1—C29—C34—C33	179.0 (4)
C16—C17—C18—C13	−0.8 (6)		

### Hydrogen-bond geometry (Å, °)

**Table:**

*D*—H···*A*	*D*—H	H···*A*	*D*···*A*	*D*—H···*A*
N1—H1A···O1^i^	0.86 (2)	1.89 (2)	2.699 (4)	156 (3)
O4—H4···O2^i^	0.82	1.66	2.460 (3)	164

Symmetry codes: (i) *x*−1, *y*, *z*.
